# Supplementary material for: Deterioration characteristics of cement-improved loess under dry–wet and freeze–thaw cycles
Source: PLoS One. 2021 Jul 1;16(7):e0253199. doi: 10.1371/journal.pone.0253199 (PMC8248616; doi:10.1371/journal.pone.0253199)
Supplement: Attachment — Submitted filename: renamed_1a283.docx [file pone.0253199.s001.docx]

**Review comments**

This paper studies the strength degradation of Cement Improved Loess formed by vertical vibration method under dry-wet and freeze-thaw cycles, which has good logic and detailed test data. The research topic has a certain engineering application value. Suggestions:

1) The research status of Cement Improved Loess at home and abroad needs to be further understood and mastered.

2) The fitting equations in Fig. 2 and Fig. 3 are best presented in tabular form.

3) The meaning of R^2^ in Fig. 2 and Fig. 3 needs to be explained.

4) The quality of English needs improving.

5) Language and writing should be concise.

6) The format shall meet the requirements of periodical format.

7) To facilitate readers to understand the test process, please add test photos of VVCM and QSCM.

8) Please add some results analysis for table 3 and table 4.
