# Supplementary material for: Deterioration characteristics of cement-improved loess under dry–wet and freeze–thaw cycles
Source: PLoS One. 2021 Jul 1;16(7):e0253199. doi: 10.1371/journal.pone.0253199 (PMC8248616; doi:10.1371/journal.pone.0253199)
Supplement: Attachment — Submitted filename: Response to Reviewers.docx [file pone.0253199.s002.docx]

**Response to Reviewers**

Dear editor:

On behalf of my co-author, I would like to thank the editorial department and the reviewers for their positive and constructive opinions and suggestions on our topic " Deterioration Characteristics of Cement-Improved Loess under Dry–wet and Freeze–thaw Cycles" (PONE-D-21-11948). We have carefully studied these suggestions and made some amendments to the paper. We have tried our best to revise the manuscript as required. After carefully considering the opinions of the editor and the reviewers, the main corrections and answers to the reviewers are as follows:

Reviewer #1: In this paper, the strength degradation of Cement Improved Loess formed by vertical vibration method under dry-wet and freeze-thaw cycles is studied, and the degradation equation and degradation coefficient are proposed. The overall structure is complete, with good logic and detailed test data. But there are several problems：

1) Some sentences need to be considered, such as vertical vibration compaction method (VVCM) molding cement improved loess, the reliability of the method is evaluated, what method is evaluated?

To solve this problem, the words used in this paper are adjusted and modified, and the reliability of the VVCM is evaluated.

2) The research status of Cement Improved Loess at home and abroad needs to be further understood and mastered.

Introduction of this paper has been rewritten and more international papers are added.

3) Describe about the experimental case study in more detail.

Modified.

4) The graphic information of vertical vibration compactor has been reflected in the corresponding references. Is it necessary to add it?

For the sake of the integrity of the paper, the idea of my collaborators and I is that we need to add.

5) Some English abbreviations appearing for the first time in this paper need to be described in detail.

Modified.

6) Part of the language needs to be further refined.

Modified.

7) The data points in Fig. 2 and Fig. 3 should be further analyzed and explained.

After data processing, the graph is analyzed and explained in depth, see 3.1 and 3.2.

8) The format of the paper should be arranged according to the requirements of the journal.

Modified.

9) The analysis of the results and the discussion part is not deep enough and needs further analysis.

After data processing, the graph is analyzed and explained in depth, see 3.1 and 3.2.

Reviewer #2: This paper studies the strength degradation of Cement Improved Loess formed by vertical vibration method under dry-wet and freeze-thaw cycles, which has good logic and detailed test data. The research topic has a certain engineering application value. Suggestions:

1) The research status of Cement Improved Loess at home and abroad needs to be further understood and mastered.

Modified. The section 1 has been rewritten.

2) The fitting equations in Fig. 2 and Fig. 3 are best presented in tabular form.

My collaborators and I think that the fitting formula is more intuitive when it is placed on the edge of the curve.

3) The meaning of R2 in Fig. 2 and Fig. 3 needs to be explained.

Modified. Where R^2^ is the coefficient of determination. The R^2^ is between 0 and 1, and the closer to 1, the better the regression fitting effect.

4) The quality of English needs improving.

Modified.

5) Language and writing should be concise.

Modified.

6) The format shall meet the requirements of periodical format.

Modified.

7) To facilitate readers to understand the test process, please add test photos of VVCM and QSCM.

As the experiment was finished, no photos were taken at that time.

8) Please add some results analysis for table 3 and table 4.

Modified.

Reviewer #3: In this manuscript, based on dry–wet cycle and freeze–thaw cycle tests, the deterioration characteristics of mechanical strength for cement-improved loess has been studied. This work is interesting. However, the studies are very poor and low. Moreover, there are many serious defects in this paper.

The main comments are as follows,

1. The English is very poor and there are many language errors. I think it should be edited by one native speaker before it is submitted.

Modified.

2. The section 1 is very bad and should be rewritten. The previous studies should be analyzed more comprehensively and deeply. And the objective of this paper should be provided clearly.

Modified. The section 1 has been rewritten.

3. Line 89, what is SCL?

Mistakes in writing. Modified.

4. Line 91, what is CSL?

Mistakes in writing. Modified.

5. Line 95, what is VVTE?

Modified. VVTE is the abbreviation of vertical vibration testing equipment.

6. Line 98, I do not know why those References have been provided here.

The value of VVTE parameter in this paper is directly used according to the research results of the research group, so we need to quote these articles.

7. Lines 83 and 102, I think the specifications can be provided as the References.

Modified.

8. Line 127, "for the specified number of rimes". is it right?

Modified.

9. Line 144, there is a wrong. "Error! Reference source not found.."

This problem caused by the wrong use of cross references in writing have been corrected.

10. In subsection 3.1.1, I think the test results should be described clearly before the table 3.

Analysis of test results has been added in subsection 3.1.1.

11. Lines 223-224, "The deterioration coefficient of CIL after the freeze–thaw cycle can be calculated using equation (2)." is it right?

Yes, it’s right.

12. Line 240, "Based on Table 3,", is it right?

Modified.

13. The studies in section 3 are too low and simple. I think deep analysis should be conducted and the test results should be verified.

Modified.

14. There are only four international papers in the Reference list. For one international manuscript, I think more international papers should be included in the Reference list.

International papers have been added.

Sincerely Yours,

Chenyang Ni
